# The impact of nitrogen deposition on photobiont‐mycobiont balance of epiphytic lichens in subtropical forests of central China

**DOI:** 10.1002/ece3.5803

**Published:** 2019-11-06

**Authors:** Ming Wang, Chuanhua Wang, Raozhen Jia

**Affiliations:** ^1^ Engineering Research Center of Eco‐environment in Three Gorges Reservoir Region Ministry of Education China Three Gorges University Yichang China; ^2^ Hubei International Scientific and Technological Cooperation Center of Ecological Protection and Management in the Three Gorges Area China Three Gorges University Yichang China; ^3^ China Three Gorges University Yichang China

**Keywords:** ammonium, chlorophyll, ergosterol, lichen, nitrate

## Abstract

Excessive nitrogen (N) deposition can impact lichen diversity in forest ecosystems, and this is a particular situation in China. Here, we examined the N uptake, assimilation, and the impact of excessive N deposition on the symbiotic balance of dominant epiphytic lichens in the subtropical forests in the Mts. Shennongjia of central China. The results show that lichen species took up, assimilated and utilized more ammonium than nitrate in a species‐specific way, following the increase of N availability. The photobiont of the lichens decreased with the increase of N concentration following an initial increase, while the mycobiont response to the N addition was not apparent. Considerable variation in response to excessive N deposition exists among the lichen species. *Usnea longissima* could regulate its N uptake, resulting in a stable photobiont‐mycobiont ratio among N treatments. In contrast, the photobiont‐mycobiont ratio of other four lichens increased initially but decreased when N concentration exceeded a certain level, and N stress may have broken the balance between photobiont and mycobiont of these lichens. Our results suggest that most epiphytic lichens in subtropical forest of central China could uptake and assimilate more ammonium than nitrate and that the balance between photobiont and mycobiont of many epiphytic lichens might change with the increasing N deposition load, which could impact the lichen diversity of this forest ecosystem.

## Graphical abstract

We found that the dominant epiphytic lichens uptake and utilization more ammonium than nitrate in a species‐specifically, which suggested that most lichens would be sensitive to nitrogen deposition. Furthermore, we found nitrogen addition would destroy the balance between mycobiont and photobiont, in detail, below the nitrogen threshold of lichens, N addition promotes the N uptake of all lichens and then increases the photobionts and promotes individual growth. When the N addition exceeds the threshold, toxic effects appear in which excess N leads to the imbalance of symbionts, resulting in a decrease in the growth rate of these lichens. Specifically, we found that *Usnea longissima* can keep a consistent balance between mycobiont and photobiont when nitrogen availability increased, which suggested that nitrogen deposition would change the lichen diversity of the subtropical forests of China.

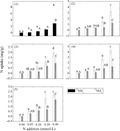

## 1 INTRODUCTION

Long‐term exposure to excessive nitrogen (N) from the atmosphere has been reported to result in a range of impacts on forest ecosystems (Aber et al., 2003). Many plant species in terrestrial ecosystems, such as some lichen species, have evolutionarily adapted to a low N level (Vitousek et al., 1997). Consequently, the excessive N deposit can negatively affect the growth and survival of plants due to the interruption of normal metabolic processes, which is particularly true for the species with low N tolerance (Bukovinszky, Frank van Veen, Jongema, & Dicke, 2008; Camargo & Alonso, 2006; Vitousek et al., 1997). Recently, the average wet N deposition load in China's forests has reached 16.6 kg hm^−2^ year^−1^, with nitrate N accounting for 37% and ammonia for 63% (Fang et al., 2011). In general, nitrogen threshold for N‐sensitive lichen species ranges from 3.1 to 4.0 kg hm^−2^ year^−1^ (Dobben & Bakker, 2013; Fenn et al., 2008), indicating that the diversity of lichens in China's forest ecosystem might have been impacted by the excessive atmospheric N deposition.

Although Lichens can take ammonium, nitrate, and organic nitrogen for their growth, NH_4_^+^ generally is the preferred form in many studies (Dahlman, Näsholm, & Palmqvist, 2010; Dahlman, Persson, Palmqvist, & Näsholm, 2004; Palmqvist & Dahlman, 2006). Since NH_4_^+^ may be more physiologically harmful than NO_3_^−^ (Britto & Kronzucker, 2002), differences of N sensitivity among species are partly attributed to with their N preference (Johansson, Nordin, Olofsson, & Palmqvist, 2010). Lichens more tolerant to N stress demonstrate a species‐specific ability to avoid excessive N uptake (Johansson et al., 2010). The exceeded nitrogen directly damages the integrity of fungal cell membranes (Munzi, Pirintsos, & Loppi, 2009), degrades algae chlorophyll (Munzi, Pisani, & Loppi, 2009), and impacts the function of photosystem II(PSII) of the algae. Indirectly or chronically, such nitrogen toxicity may lead to a carbon imbalance in lichen symbionts (Palmqvist, 2000), which is reflected in the symbiotic imbalance between algae and fungi (Palmqvist & Dahlman, 2006).

Previous studies suggest that the healthy growth of lichens is a consequence of their well‐balanced symbiosis of photobiont and mycobiont (Johansson, Olofsson, Giesler, & Palmqvist, 2011), which had been accepted as a fundamental mechanism in the maintenance of the lichen diversity (Honegger, 2003). Studies also showed that the response of lichen symbiosis of photobiont and mycobiont to N stress may be species‐specific. For example, nitrogen addition inhibits fungal expansion in *Nephroma arcticum*, increase the total biomass and the proportion of the photosynthetic algae synchronously. The same scenario also was reported in another N‐sensitive lichen, *Evernia prunastri* (Gaio‐Oliveira, Dahlman, Palmqvist, Martins‐Loução, & Máguas, 2005). However, evidence also exists that N improves the photosynthetic capacity of photobionts, and subsequently promotes the growth of several lichen species (Palmqvist & Dahlman, 2006). Such mixed response indicates that N deposition could impact the growth and survival of the lichens in a species‐specific way. In general, it is plausible that lichens may take up more ammonium, and the imbalance between algae and fungi induced by N deposition stress could be a main cause driving some lichen species to extinction (Gaio‐Oliveira, Branquinho, Máguas, & Martins‐Loução, 2001; Smith & Griffiths, 1996).

It has been reported that many epiphytic lichens in the subtropical forests of central China are very sensitive to N deposition (Guo, Wang, & Yang, 2016; Wang, Yang, Yuan, Munzi, & Liu, 2015). Besides the ecological roles in forests system, epiphytic lichens in this region are particularly important as they are important overwinter food resources for two critically endangered snub‐nosed monkey species endemic to China (*Rhinopithecus roxellana* and *Rhinopithecus bieti*) (Li, 2006; Liu, Stanford, Yang, Yao, & Li, 2013; Tie et al., 2010). However, some critical issues concerning the mechanisms if N uptake differed in N forms and its effects on symbiotic balance of the epiphytic lichens in the subtropical forests of central China have not been addressed.

In this study, we hypothesized that the epiphytic lichens in subtropical mountains of central China would uptake and utilize more ammonium than nitrate, which may subsequently break the balance between photobionts and mycobionts of the lichens in a species‐specific way. By using five dominant epiphytic lichens in this region, we addressed the following questions: (a) Do epiphytic lichens in the subtropical mountains of the central China selectively uptake and assimilate ammonium and nitrate? (b) Does nitrogen addition influence the photobiont and mycobiont balance of lichen species? and whether different species reacts differently, with a species‐specific way, if that is the case? Our findings will help to understand the mechanism of N stress on lichen diversity, and provide experimental evidence on the effects of increased nitrate on lichen diversity. Such a study is critically important in conveying an important information to the public when envisaging the deteriorated environment and the air pollution in China.

## 2 MATERIALS AND METHODS

### 2.1 Study site

The study site in Mts. Shennongjia, the northwestern part of Hubei Province, China (30°15′N–31°57′N, 109°56′E58′–110°E), is featured with warm and moist weather, with an annual precipitation of 1,350 mm and annual mean temperature of 10.6°C (see Chen et al., 2019; Chen, Zhong, Sun, Xie, & Zhou, 2017). The total wet N deposition of the study site is ca. 11.89 kg hm^−2^ year^−1^, of which ammonium accounts for 44.08%, nitrate for 40.75%, and nitrite for 15.17%, respectively (Yang, Deng, Guo, Xu, & Wang, 2017). The vegetation of this region principally includes deciduous broadleaved mixed forest and coniferous forest, with yellow brown soil (Tie et al., 2010; Zhang et al., 2014).

### 2.2 Materials

We focused on five dominant epiphytic lichens in native forests of the region, including *Usnea longissima*, *Usnea luridorufa*, *Usnea dasopoga*, *Usnea betulina*, and *Ramalina calicaris* var. *japonica* (Wu, 1997; Yang et al., 2017). The study materials of these lichens were collected from one tree species *Salix wallichiana* in a gorge at the study site (31°34′N, 110°23′E, at an altitude of 1,700 m). Healthy lichens were collected and subsequently dried at room‐temperature before stored in a freezer (−18°C) for later experiments.

### 2.3 Nitrogen treatments exploring the differences in the adsorption of ammonium and nitrate by the lichens

Before N treatments, all lichens were activated following the method as described in Palmqvist (1993). Briefly, lichens were sprayed with distilled water and activated in a chamber for 48 hr under 15°C for 12 hr of light and 12 hr of dark (PAR 150 μmol m^−2^ s^−1^) regimes.

To explore the differentiated adsorption efficiency of ammonium and nitrate among lichen species, a random experiment was conducted following the methods of Dahlman et al. (2004). The activated lichens were placed in a transparent cup containing 30 ml 1.0 mmol/L NH_4_NO_3_ solution labeled by either ^15^NH_4_^+^ or ^15^NO_3_^−^. For a given lichen species, two treatments (^15^NH_4_^+^ and ^15^NO_3_^−^) and five replicates were carried out. Lichen thalli were completely submerged in the solution and incubated at 15°C in a completely dark chamber. During the submersion, an air pump was used to ensure the solution was oxygenated. Lichens were removed after 30 min and rinsed with 1.0 mmol/L CaCl_2_ twice to clean the N markers adsorbed on cell channels. The treated lichen thalli were oven‐dried at 60°C for 24 hr and stored at −80°C.

### 2.4 Nitrogen treatments exploring differences in the utilization of ammonium and nitrate by the lichens

This treatment was carried out in the laboratory. First, 0.00, 0.05, 0.10, 0.20, and 0.40 mmol/L NH_4_NO_3_ were used to simulate increasing N loads (note the annual average N concentration in the Mts. Shennongjia is ca. 0.097 mmol/L, Yang, Wang, & Wang, 2018). The N solution was prepared with artificial rainwater containing 8.8 mg/L K_2_CO_3_, 4.6 mg/L Na_2_CO_3_, 5 mg/L CaCO_3_, 4.4 mg/L ^1^NaH_2_PO_4_, 0.25 mg/L Fe_2_SO_4_·7H_2_O, and 0.6 mg/L MnSO_4_H_2_O (Tamm, 1953). After that, ^15^NH_4_NO_3_ or NH_4_^15^NO_3_ were used to label the solutions separately. Eventually, the concentration of ^15^N in the solution was adjusted to 0.002 mmol/L. For a given lichen species, ten transparent plastic boxes with a diameter of 6.5 cm were used to harbor lichen thalli. In each box, two layers of filter paper were placed, and four activated lichens were then randomly placed on the filter paper. Each box was randomly treated with one of the ten labeled N solutions, and a mini atomizer sprayer was used twice a week. Lichens were cultivated in a chamber at 15°C under a 12 hr light and 12 hr dark (PAR150 μmol m^−2^ s^−1^) regime for 2 months (Gaio‐Oliveira, Dahlman, Palmqvist, & Maguas, 2004). Lichens were then oven‐dried at 60°C for 24 hr, and stored at −80°C for later use.

### 2.5 Nitrogen treatment assessing the impact of N addition on the balance between algae and fungi in the lichen

This test was carried out in the laboratory with the cultivation method described in Section 2.4. Artificial rainwater solutions (composition as described in above section) with N concentrations of 0.00, 0.05, 0.10, 0.20, and 0.40 mmol/L without ^15^N markers were used to treat lichen thalli (Tamm, 1953) with a duration of 60 days (Gaio‐Oliveira et al., 2004). After that, each thallus was cut into two parts: One was used to determine chlorophyll content after air‐dried, and the other was dried at 60°C for 48 hr in the oven for later determination of ergosterol content.

### 2.6 Chemical analysis

Total N concentration and isotopic ratio of ^15^N:^14^N were analyzed by the Agricultural Environmental Stable Isotope Laboratory (AESIL, Chinese Academy of Agricultural Sciences, China) with the elemental analyzers (vario PYRO cube, Elementar), and mass spectrometers (Isoprime 100, Isoprime). The amount of N uptake was calculated with the following equation:

$$N\;\text{uptake}=\left[\text{(At}{\%}_{S}-\;\text{At}{\%}_{C})\times {\text{TN}}_{S}\right]/R^{15}N$$

where At%_S_ is ^15^N relative atomic percentage of the N‐treated sample; At%_C_ is ^15^N relative atomic percentage of the control; TN_S_ is N content of the treated sample (g/g); R^15^N is the proportion of ^15^N content in the N solution (Johansson et al., 2010).

Chlorophyll pigments were quantified with the method described by Barnes, Balaguer, Manrique, Elvira, and Davison (1992). Ten mg lichen fragments were rinsed with 3 ml acetone saturated with CaCO_3_ for four times and five min for each time. Then the fragment was placed into 5 ml dimethyl sulfoxide solution containing 2.5 mg/L polyvinylpyrrolidone. After incubation at 65°C for 40 min, the solution absorbance was determined at wavelengths of 665 and 648 nm with an ultraviolet spectrophotometer (UV5000, Shanghai Metash Instruments Co. Ltd). The total concentration of chlorophyll (*C*_a+b_) was calculated with the following formula:

$$C_{a+b}=7.49A_{665}+20.34A_{648}.$$

Ergosterol was quantified by three‐wavelength spectrophotometry method (Guo, Luo, Chen, & Zeng, 1983). Ergosterols have a larger peak absorption at 282 nm and two small ones at 272 and 292 nm. Thus, the regression between ∆*A* (∆*A* = *A*_282_ − (*A*_272_ + *A*_292_)/2) and ergosterol content (*ρ*) was established as the following: *A* = 5.7196*ρ* − 0.0005 (*R* ^2^ = 0.9991). Following the methods of Guo et al. (1983), ergosterol was extracted from lichen thalli. An amount of 0.50 g dry lichen was placed in a tube with 16 ml of 25% alkali‐alcohol solution (25 g KOH was dissolved in 40 ml distilled water, and then supplemented with 100 ml anhydrous ethanol). Tubes were incubated in a water bath at 90°C for 2.5 hr. Subsequently, two mL anhydrous ethanol was added into the tubes that were incubated for a further 1.5 hr for saponification. Ten mL of N‐heptane was added and oscillated for approximately 30 s. The top N‐heptane solution was then evaporated with a rotary evaporator. Finally, the extract was dissolved with 10 ml alcohol so that its absorption levels at 272, 282, and 292 nm were consequently determine.

### 2.7 Data analysis

For a given species, one‐way ANOVA was used to analyze the differences of N absorption and utilization, between nitrate and ammonium, and the changes of the algae to fungi proportion among N levels. Significance level taken at *p* < .05. Statistical analysis was implemented with SPSS 13.0 (IBM Corp.). Figures were plotted with Origin 8.0.

## 3 RESULTS

### 3.1 Species‐specific adsorption of ammonium and nitrate

There were significant interspecies differences in N content and percentage of ^15^N among the five lichen species (Table 1). Nitrogen content was highest in *U. longissima* (1.32 ± 0.02%), while it was lowest in *U. betulina* (0.79 ± 0.16%).

**Table 1:** Nitrogen contents in the lichens, and nitrogen taken by the lichens treated with different levels of N addition

Lichen species	Total nitrogen (%)	^15^N percent (%)	Uptake of N‐NH_4_^+^ (mg g^−1^ min^−1^)	Uptake of N‐NO_3_^−^ (mg g^−1^ min^−1^)	N‐NH_4_^+^/N‐NO_3_^−^
*Usnea longissima*	1.3241 ± 0.0185^c^	0.3602 ± 0.0001^a^	0.3611 ± 0.0426^c^	0.0050 ± 0.0006^a^	71.62
*Usnea luridorufa*	0.9980 ± 0.0449^b^	0.3615 ± 0.0001^b^	0.3074 ± 0.0533^bc^	0.0065 ± 0.0014^ab^	46.94
*Ramalina calicaris* var. *japonica*	0.9001 ± 0.1098^ab^	0.3621 ± 0.0005^b^	0.1512 ± 0.0121^a^	0.0057 ± 0.0020^ab^	26.68
*Usnea dasopoga*	0.8478 ± 0.0729^ab^	0.3610 ± 0.0002^ab^	0.1880 ± 0.0497^ab^	0.0200 ± 0.0072^c^	9.38
*Usnea betulina*	0.7902 ± 0.1590^a^	0.3611 ± 0.0013^ab^	0.5269 ± 0.2084^d^	0.0119 ± 0.0071^b^	44.43

Note

Data in columns are the mean ± *SD* (*n* = 4). Different letters in the columns denote significant differences between species.

The results showed that ammonium taken by the five lichen species are from 9.4 to 71.6 times higher than the nitrate, suggesting that all lichen species more preferred ammonium to nitrate. The results also showed that the amount of both ammonium and nitrate taken up significantly differed among the lichen species (Table 1). *Usnea betulina* had the highest capacity of adsorbing the ammonium, approximately 3.5 times higher than that of the species with the lowest capacity, *R. calicaris* var. *japonica*. The amount of the nitrate uptaken by *U. dasopoga* (0.02 ± 0.01 mg g^−1^ min^−1^) was twice higher than by *U. longissima* (0.01 ± 0.00 mg g^−1^ min^−1^).

### 3.2 Species‐specific utilization of ammonium and nitrate

**Figure 1:**
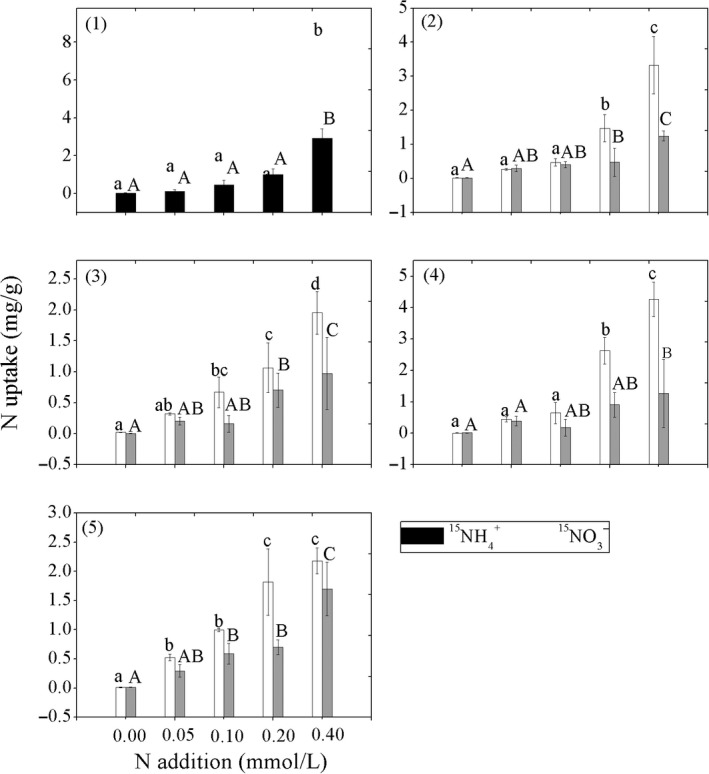
Differentiation of nitrogen taken by the epiphytic lichens under nitrogen stress. *White bars* ^15^NH_4_^+^ uptake; *gray bars* ^15^NO_3_^−^ uptake; (1) *Usnea longissima*; (2) *Usnea luridorufa*; (3) *Ramalina calicaris* var. *japonica*; (4) *Usnea dasopoga*; (5) *Usnea betulina*. Lower and uppercase letters on the bars indicated significant differences between NH_4_^+^ or NO_3_^−^ addition treatments, respectively

All the five species utilized 3.88 times more ammonium than nitrate. *Ramalina calicaris* var. *japonica*, *U. dasopoga*, and *U. betulina* utilized more nitrate than other two species under lower N availability conditions (Figure 1). Nitrogen utilization, both ammonium and nitrate components, of the five lichen species generally increased with the increased N availability. When N availability increased to 0.20 mmol/L, the ammonium utilized by *U. longissima*, *U. luridorufa*, *R. calicaris* var. *japonica*, *U. dasopoga*, and *U. betulina* were 0.27, 3.13, 1.60, 4.11, and 1.82 times, respectively, higher than that at the control level of 0.10 mmol/L (equivalent to the background N concentration of 0.097 mmol/L at the region). The ammonium used by the four species increased to 4.42, 7.06, 2.91, 6.66, and 2.17 times higher compared to that under controlled level when N availability increased to 0.4.0 mmol/L. Similarly, compared to the control (0.1 mmol/L), the nitrate utilized by the five lichen species was 2.17, 1.19, 4.39, 5.11, and 1.19 times higher when N availability reached 0.40 mmol/L, and the nitrate availability is 6.34, 3.09, 6.09, 7.18, and 2.88‐time higher (Figure 1).

### 3.3 Impacts of N stress on the balance of photobionts and mycobionts in epiphytic lichens

**Figure 2:**
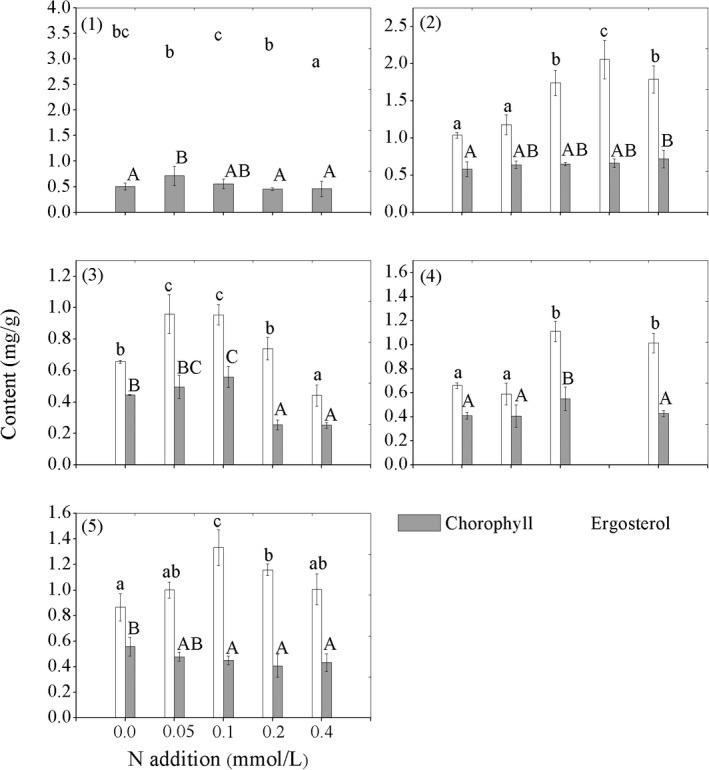
Response of the photobiont and mycobiont of five epiphytic lichen species to nitrogen stress. *White bars* ^15^NH_4_^+^ uptake; *gray bars* ^15^NO_3_^−^ uptake; (1) *Usnea longissima*; (2) *Usnea luridorufa*; (3) *Ramalina calicaris* var. *japonica*; (4) *Usnea dasopoga*; (5) *Usnea betulina*. Lower and uppercase letters on the bars of the lichens indicated significant differences between NH_4_^+^ or NO_3_^−^ addition treatments, respectively

A comparison of the contents of chlorophyll and ergosterol, which represents the balance of photobionts and mycobionts, among the five lichen species in response to the simulated N additions is provided in Table 2 and Figure 2. Except for *U. longissima*, the chlorophyll contents of the other four species increased initially with N addition, then decreased with higher N availability at a certain threshold. The N availability thresholds for the four lichen species indicated by chlorophyll were close to 0.20 mmol/L, with *U. luridorufa* at 0.4 mmol/L, implying *U. luridorufa* is more tolerant to N stress than other four species (Figure 2). Meanwhile, the mycobiont, indicated by the ergosterol content of the five species, was less impacted by N addition. Compared with the treatment at 0.10 mmol/L, mycobionts of *R. calicaris* var. *japonica* and the *U. dasopoga* decreased with increased N addition. The other three species, however, were not impacted by the increased N addition. Overall, we found that N addition significantly impact the balance between the photobiont and mycobiont of four lichen species except *U. longissima* (Table 2).

**Table 2:** The influence of nitrogen addition to the ratio of chlorophyll and ergosterol in the lichens

N addition (mM/L)	Lichen species				
	*Usnea longissima*	*Usnea luridorufa*	*Ramalina calicaris* var. *japonica*	*Usnea dasopoga*	*Usnea betulina*
0.00	6.15 ± 0.58^a^	1.84 ± 0.20^a^	1.47 ± 0.01^a^	1.62 ± 0.03^a^	1.55 ± 0.01^a^
0.05	4.33 ± 0.81^a^	1.84 ± 0.08^a^	1.97 ± 0.21^a^	1.49 ± 0.15^a^	2.11 ± 0.14^ab^
0.10	5.92 ± 0.45^a^	2.69 ± 0.17^b^	1.72 ± 0.06^a^	2.08 ± 0.25^b^	2.97 ± 0.22^c^
0.20	6.45 ± 0.28^a^	3.14 ± 0.26^b^	2.94 ± 0.26^b^	Not available	2.92 ± 0.25^c^
0.40	6.20 ± 1.34^a^	2.57 ± 0.30^b^	1.75 ± 0.12^a^	2.37 ± 0.12^b^	2.39 ± 0.32^bc^

Note

Data in columns are the mean ± *SD* (*n* = 4). The lowercase letters in the columns denote significant differences between species.

## 4 DISCUSSION

### 4.1 Selective N uptake and utilization of the epiphytic lichens in the Mts. Shennongjia

Our results show that all the five lichen species uptake more ammonium than nitrate, and there is a significant differentiation between the species (see Table 1). This is consistent with the results found in 13 lichen species in Sweden (Dahlman et al., 2004). In general, lichens uptake ammonium via a passive path, requiring less energy than taking up nitrate (Munzi, Pirintsos, et al., 2009; Munzi, Pisani, et al., 2009). Furthermore, ammonium can directly participate in N assimilation in lichens (Brown, Avalos, Miller, & Bargagli, 1994; Chapin, Bloom, Field, & Waring, 1987), so that the cost of ammonium assimilation is lower than that of nitrate. Compared to the results of Dahlman et al. (2010), the amount of ammonium in the epiphytic lichens in Mts. Shennongjia is much higher than that in the European region, which may be attributed to their species‐specific N uptake abilities. Meanwhile, the average N content of the five epiphytic lichen species studied here is 9.72 mg/g, which is also higher than that of *Alectoria sarmentosa* and *Platismatia glauca* (5.5 mg/g, Johansson et al., 2010). Such a phenomenon could be attributed to the comprehensive effect of the increased N deposition levels in the Mts. Shennongjia following a deteriorated environment (Yang et al., 2018). Our results further consolidated the findings that most of epiphytic lichen species in this region is very sensitive to excessive N deposition (Guo et al., 2016; Wang et al., 2015).

We also found that N addition has significantly increased N utilization, especially taken up more ammonium by all the five epiphytic lichen species (Figure 1), which is consistent with the results reported by Palmqvist & Dahlman (2006). Previous studies suggested that N‐intolerant epiphytic lichens accumulate more N when the availability increased, which is assumed as a mechanism of N‐intolerant lichen species under N stress (Gaio‐Oliveira et al., 2005; Hogan, Minnullina, Sheppard, Leith, & Crittenden, 2010). Interestingly, Wang et al. (2015) found that different N form has species‐specific impact on the health of the four epiphytic lichens (*U. longissima*, *U. betulina*, *R. calicaris* var. *japonica*, and *U. dasopoga*), and that nitrate also impacts cell membrane integrity and photosynthesis organs of the species. Compared to the average nitrate proportion (37%) of the total nitrogen deposition in China's forests system (Fang et al., 2011), forests system at Mts. Shennongjia has a slightly higher nitrate at 43%. Given the less toxicity of the nitrate, a proportional increase in nitrate in N deposition could mitigate the adverse impact of nitrogen deposition on lichen diversity at Mts. Shennongjia region, which warrants further studies.

### 4.2 Impacts of N addition on the balance of photobionts and mycobionts of the lichens in Mts. Shennongjia

Nitrogen addition significantly increases chlorophyll content in our study, which is consistent with the results from Johansson et al. (2010). Chlorophyll content in epiphytic lichens is positively correlated with photosynthetic capacity so that chlorophyll content is often used to indicate the proportion of photosynthetic organisms in the lichens (Palmqvist et al., 2002; Raven, Johnston, Handley, & Mcinroy, 2010). With the increased of external N, more N resources obtained by the lichens are deposited in photosynthetic organisms, which can, in turn, increase carbon acquisition of the lichens (Palmqvist et al., 2002). However, when the concentration of N exceeds the tolerance for the lichen, chlorophyll composition of the lichens could be degraded, resulting in the decreased chlorophyll content (Guo et al., 2016). Therefore, to some extent, we can assess N stress faced by the sensitive lichens by evaluating the fluctuation of chlorophyll contents with N addition treatments.

Ergosterols are important components of the fungal plasma membrane and are used to indicate the proportion of fungi in the lichens (Gaio‐Oliveira et al., 2005). In this study, the responses of ergosterol contents in *U. longissima*, *R. calicaris* var*. japonica*, and *U. dasopoga* to N addition are consistent with chlorophyll responses (Figure 2), but the magnitudes of changes in ergosterol are smaller than that in chlorophyll. In contrast, ergosterol contents of *U. luridorufa* and *U. betulina* change less under lower levels of N stress (Figure 2). A possible explanation for this phenomenon may be the fact that mycobiont growth is not limited by N (Johansson et al., 2011). Destruction of the balance between lichen photobiont and mycobiont induced by N deposition is an important mechanism leading to the loss of lichen diversity reported in Europe (Johansson et al., 2011). In our previous investigation, we found that propagule survival of the *U. longissima* is not efforted by N addition when the load is less than 57.99 kg N ha^−1^ year^−1^. In contrast, the other four species showed higher sensitivity to nitrogen addition (Jia, Wang, & Wang, 2019; Wang, Munzi, Wang, Jia, & Wang, 2019). In this research, we found that balance of the chlorophyll and the ergosterol in the *U. longissima* under N stress is relatively stable, though it assimilates more ammonium and nitrate than the other four species (Table 2), indicating that it may have a stronger regulatory ability to alleviate toxic effect of the N stress. In contrast, the balance between algae and fungi is impacted in the other four species and the impact increased following the increased N stress (Figure 2 and Table 2). Thus, our findings further consolidate the theory that some lichens are capable of stabling the balance of the mycobiont‐photobiont, which is vital in conserving the diversity of the lichens under N stress (Johansson et al., 2011).

### 4.3 Conservation of lichen diversity in central China

Because of its intact evergreen and deciduous broad‐leaf mixed forests and refuge for ancient relic wildlife, Mts. Shennongjia have been regarded as one of the most important subtropical forest systems in central China (Wang et al., 2017). Our results suggest that the dominant epiphytic lichens in this region have a higher affinity to the ammonium, implying that the lichens in this region could be negatively impacted by the increased ammonium. Lichen species in our study responded to N stress in a species‐specific way, with *U. longissima* is uniquely featured by its relative stronger N tolerance than the other four species. More attention therefore should be paid to the studies associated with the N pollution, especially aero ammonium, and the frequent surveying on lichen diversity of subtropical forest system in central China. The results in this study also suggest that photobiont of all the five lichen species increases with the increased N availability, which may benefit to the maintenance of lichen symbiosis. Thus, it is likely that ratio of the photobiont to mycobiont of the lichens is a useful bio‐mark in monitoring the impact of N deposition and lichen diversity in central China as this ratio is sensitive to N loads.

## 5 CONCLUSIONS

The dominant epiphytic lichens in subtropical mountains in central China took up and utilize more ammonium than nitrate, which could be a key mechanism underlying the sensitivity of epiphytic lichens in subtropical zones. Algae and fungi of the five lichen species studied respond to N addition in a species‐specific way. The excessive N addition leads to a photobiont and mycobiont imbalance for the lichens. This could eventually impact lichen diversity of the region.

## CONFLICT OF INTEREST

No conflict of interest exits in the submission of this manuscript.

## AUTHOR CONTRIBUTIONS

In this work, Dr. Chuanhua Wang finished the protocol of the experiment and the manuscript, also participated in writing of manuscript; Mr. Ming Wang conducted the experiment, and finished most part of the manuscript; Miss Raozhen Jia collaborated with Mr. Ming Wang to conduct the experiment.

## Data Availability

The data will be made available in the Dryad Digital Repository upon acceptance of the manuscript (https://doi.org//10.5061/dryad.rn8pk0p5j). The authors are solely responsible for these data. Queries (other than absence of the material) should be directed to the corresponding author.
